# Association of the Estimated 24-H Urinary Sodium Excretion with Albuminuria in Adult Koreans: The 2011 Korea National Health and Nutrition Examination Survey

**DOI:** 10.1371/journal.pone.0109073

**Published:** 2014-10-14

**Authors:** Sang Youb Han, Jae Won Hong, Jung Hyun Noh, Dong-Jun Kim

**Affiliations:** Department of Internal Medicine, Ilsan-Paik Hospital, College of Medicine, Inje University, Goyang, Republic of Korea; University of São Paulo School of Medicine, Brazil

## Abstract

**Background:**

Sodium intake and albuminuria have important roles in blood pressure and renal progression. Although their relationship has been reported, the results have not been consistent and all studies have examined small populations.

**Objective:**

This study investigated the role of the estimated 24-h urinary sodium excretion as a marker of sodium intake and albuminuria.

**Design:**

This investigation included 5,187 individuals age 19 years and older from a cross-sectional, nationally representative, stratified survey: The Korea National Health and Nutrition Examination Survey (KNHANES V-2), in 2011. Albuminuria was defined as a urinary albumin/creatinine ratio ≥30 mg/g. The 24-h urinary sodium excretion was estimated from a spot urine.

**Results:**

On classifying our participants into quartiles based on the estimated 24-h urinary sodium excretion, the prevalence of albuminuria increased with the 24-h urinary sodium excretion (5.3, 5.7, 7.5, and 11.8% in the first through fourth quartiles, respectively, *p* for trend <0.001). Even after adjusting for age, sex, diabetes, obesity, and hypertension, the significance persisted. In a multiple logistic regression analysis, the second and third quartiles of the estimated 24-h urinary sodium excretion were not associated with the presence of albuminuria with the first quartile as a control. However, the fourth quartile was significantly associated with the presence of albuminuria (odds ratio 1.61 [95% confidence interval 1.71–2.21], *p* = 0.003) after adjusting for age, sex, diabetes, obesity, and hypertension.

**Conclusions:**

These findings suggest that salt intake is associated with the presence of albuminuria in the general Korean adult population.

## Introduction

The prevalence of microalbuminuria is 5.1–13.7% in the general population according to the Third National Health and Examination Survey (NHANES III) and a Japanese general population study [1], [2]. Microalbuminuria reflects endothelial dysfunction and is a risk factor for cardiovascular complications in the general population [3], [4]. It is also an important risk factor for progression toward cardiovascular and renal events in patients with diabetes and hypertension [5], [6]. Reducing albuminuria could reduce both renal and cardiovascular events [7].

Sodium intake is associated with hypertension and rapidly declining renal function. Increased sodium intake is also related to stroke and cardiovascular mortality in a meta-analysis [8]. Regarding the role of sodium in the blood pressure and renal progression, it is now clear that reducing dietary salt consumption is a good strategy for preventing cardiovascular and renal disease progression [9], [10].

In 2011, the Korea Centre for Disease Control examined urine microalbuminuria in all participants in the fifth Korea National Health and Nutrition Examination Survey (KNHANES V-2). Previously, we reported that the weighted prevalence of microalbuminuria was 5.2% (95% confidence interval (CI) 4.4–6.1) and that of macroalbuminuria was 1.0% (95% CI 0.7–1.4) in this Korean general population and older age, female sex, diabetes, and hypertension were independently associated with the presence of albuminuria [11].

Both sodium intake and microalbuminuria have important clinical meaning in various diseases. However, the relationships are not clear. We hypothesised that a high sodium intake was associated with albuminuria. Therefore, this study investigated the association between sodium intake and microalbuminuria in a nation-wide population.

## Subjects and Methods

### Characteristics of subjects

This investigation included 5,187 individuals age 19 years and older from a cross-sectional, nationally representative, stratified survey: the Korea National Health and Nutrition Examination Survey (KNHANES). This is conducted regularly by the Korea Centre for Disease Control. A representative population was recruited using population-allocation-systematic sampling with multistage stratification. Its operation and procedure have been reported [11]. The study was approved by the Institutional Review Board of Ilsan-Paik Hospital (IB-2-1312-053). After approval of the study proposal, the KNHANES dataset was made available at the request of the investigator. Since the dataset did not include any personal information and the participants in KNHANES had already consented, our study was exempted by the board from the necessity to obtain participant consent.

### Microalbuminuria

The serum and urinary creatinine concentrations of a random sample were measured using a colourimetric method (Hitachi Automatic Analyser, Hitachi, Japan). Urinary albumin was measured using a turbidimetric immunoassay (Hitachi Automatic Analyser 7600, Hitachi, Japan). Microalbuminuria was defined as an albumin/creatinine ratio (ACR) of 30–300 mg/g and macroalbuminuria as a ratio >300 mg/g [12]. The estimated glomerular filtration rate (eGFR) was determined using the Modification of Diet in Renal Disease (MDRD) formula: eGFR (ml/min/1.73 m^2^) = 175×SCr (mg/dl)^−1.154^×Age^−0.203^×0.742 (if female) [13]. The urine albumin and creatinine concentrations were measured in the same laboratory during all surveys. The inter-assay coefficient of variation for all laboratory work was constantly low (<3.1%). The results of the detailed health interviews, physical examinations, and other laboratory procedures have been published elsewhere [11].

### Estimating the 24-h urinary sodium excretion

The 24-h urinary sodium was estimated from the sodium and creatinine of random urine samples using Tanaka’s equation [14]: 24-h urinary Na excretion (mmol/day)  = 21.98×U_Na_/U_Cr_×{–2.04×age+14.89×weight (kg)+16.14×height (cm) –2244.45}^0.392^.

### Statistical analyses

Data are presented as means and standard error (SE). Age- and sex-adjusted comparisons of the clinical characteristics according to the quartile of the estimated 24-h urinary sodium excretion were made using analysis of covariance (ANCOVA).

Logistic regression was used to analyse the association between microalbuminuria and quartile of the estimated 24-h urinary sodium excretion. The data were analysed using SPSS 10.8 for Windows (SPSS, Chicago, IL, USA).

## Results

### Comparisons of baseline characteristics according to microalbuminuria

The mean age of the study population was 52 (range 19–97) years. The prevalence of diabetes and hypertension was 11.0% and 32.4%, respectively. The prevalence of microalbuminuria and macroalbuminuria was 6.3% and 1.3%, respectively (males 5.9% and 1.4%; females 6.6% and 1.2%). After adjusting for sex and age, waist circumference, body mass index (BMI), systolic and diastolic blood pressure, serum triglycerides, fasting plasma glucose, and the proportions of obesity, hypertension, and diabetes increased with the degree of albuminuria (*P* for trend <0.05). By contrast, the eGFR and estimated 24-h urinary sodium excretion decreased with the degree of albuminuria (*P* for trend <0.01) (Table 1).

**Table 1:** Age, sex, and age- and sex-adjusted demographic and clinical characteristics of the Korean population, age 19 years and older, in the 2011 KNHANES according to albuminuria.

	NA	mA	MA	*P*
Number (weighted)	4793	328	66	
Age (years)	50.4±0.2	61.4±0.9	63.7±2.0	<0.001
Female (%)	55.0	55.0	51.5	0.492
Current smoking (%)	20.8±0.5	24.1±2.0	25.2±4.5	0.181
Heavy alcohol drinking (%)	7.2±0.4	8.2±1.4	7.1±3.1	0.768
Regular exercise (%)	13.1±0.5	9.5±1.9	16.7±4.1	0.116
Waist circumference (cm)	81.6±0.1	83.9±0.5	84.2±1.2	<0.001
BMI (kg/m^2^)	23.7±0.1	24.3±0.2	24.2±0.4	0.003
Obesity (%)	32.3±0.7	39.5±2.6	35.9±5.8	0.028
Systolic BP (mmHg)	118.8±0.2	126.6±0.8	135.3±1.9	<0.001
Diastolic BP (mmHg)	75.6±0.1	78.2±0.6	78.8±1.2	<0.001
Anti-hypertensive drugs (%)	20.2±0.5	36.2±2.0	46.7±4.5	<0.001
Hypertension (%)	30.7±0.6	52.2±2.3	63.5±5.1	<0.001
FPG (mg/dL)	97.0±0.3	107.4±1.2	127.7±2.7	<0.001
Diabetes (%)	9.5±0.4	27.1±1.7	44.9±3.7	<0.001
Serum LDL-C (mg/dL)	114.9±0.8	111.2±3.6	121.8±8.4	0.417
Serum TG (mg/dL)	132.6±1.5	152.5±5.8	189.1±12.9	<0.001
Anti-lipid drug (%)	6.5±0.4	9.4±1.4	10.3±3.0	0.068
Serum creatinine (mg/dL)	0.8±0.1	0.9±0.1	1.1±0.1	<0.001
eGFR (ml/min/1.73 m^2^)	86.8±0.2	86.3±0.8	75.5±1.8	<0.001
eGFR<60 ml/min/1.73 m^2^ (%)	3.6±0.3	9.4±1.1	29.8±2.4	<0.001
ACR (mg/g)	4.3±2.0	84.3±7.6	1025.4±16.7	<0.001
Urine Na^+^ (mmol/L)	124.9±0.7	125.7±2.9	112.6±6.3	0.148
Urine Cr (mmol/L)	13.2±0.1	12.1±0.4	11.1±0.8	0.002
U [Na^+^]/[Cr] (mmol/mmol)	12.7±0.1	14.7±0.5	15.9±1.0	<0.001
24-h urinary sodium (mg/day)	4383±40	5080±156	5440±345	<0.001

Data are expressed as means with SEM. Abbreviations: albuminuria, microalbuminuria or macroalbuminuria; NA, normoalbuminuria; mA, microalbuminuria; MA, macroalbuminuira; BMI, body mass index; obesity, BMI ≥25 kg/m^2^ or more; BP, blood pressure; hypertension, systolic blood pressure ≥140 mmHg or diastolic blood pressure ≥90 mmHg or use of antihypertensive medications irrespective of BP; FPG, fasting plasma glucose; diabetes, fasting plasma glucose ≥7.0 mmol/L or current anti-diabetes medication or a previous diagnosis of diabetes by a physician; LDL-C, low density lipoprotein-cholesterol; TG, triglyceride; eGFR, estimated glomerular filtration rate; ACR, albumin-creatinine ratio.

### Comparisons of baseline characteristics according to the estimated 24-h urine Na excretion

The group with the higher estimated 24-h urinary sodium excretion was more likely to be older, female, and obese. The BMI, blood pressure, triglycerides, and eGFR were higher in the higher quartile group (Table 2).

**Table 2:** Age, sex, and age- and sex-adjusted demographic and clinical characteristics of the Korean population, age 19 years and older, in the 2011 KNHANES based on the estimated 24-h urinary sodium excretion.

	Quartile 1(149–2360 mg/day)	Quartile 2(2361–3765 mg/day)	Quartile 3(3766–5780 mg/day)	Quartile 4(5781–31021 mg/day)	*P*
Number (weight)	1282	1300	1304	1301	
Age (years)	45.2±0.4	48.4±0.4	53.5±0.4	58.0±0.4	<0.001
Female (%)	47.5	51.7	57.0	64.3	<0.001
Waist circumference (cm)	80.0±0.3	81.5±0.3	82.1±0.3	83.5±0.3	<0.001
BMI (kg/m^2^)	23.2±0.1	24.7±0.1	23.8±0.1	24.3±0.1	<0.001
Obesity (%)	28.1±1.3	33.1±1.3	32.5±1.3	37.6±1.3	0.028
Systolic BP (mmHg)	117.3±0.4	118.3±0.4	119.5±0.4	123.0±0.4	<0.001
Diastolic BP (mmHg)	74.6±0.3	75.5±0.3	76.0±0.3	77.3±0.3	<0.001
Anti-hypertensive drugs (%)	26.3±1.0	21.4±1.0	18.4±1.0	20.3±1.0	<0.001
Hypertension (%)	33.5±1.2	31.2±1.2	30.8±1.2	34.4±1.2	0.080
FPG (mg/dL)	99.0±0.6	97.5±0.6	97.9±0.6	97.7±0.6	0.290
Diabetes (%)	11.7±0.9	9.6±0.8	11.2±0.8	11.6±0.9	0.259
Serum LDL-C (mg/dL)	116.0±1.5	114.8±1.6	115.5±1.6	112.5±1.7	0.445
Serum TG (mg/dL)	126.4±3.0	127.7±2.9	139.1±2.9	144.9±3.0	<0.001
Anti-lipid drug (%)	8.1±0.7	6.1±0.7	6.7±0.7	6.0±0.7	0.118
eGFR (ml/min/1.73 m^2^)	83.7±0.4	85.5±0.4	86.7±0.4	90.7±0.4	<0.001
eGFR<60 ml/min/1.73 m^2^(%)	3.6±0.3	9.4±1.1	29.8±2.4	144.9±3.0	0.122
Urine Na^+^ (mmol/L)	82.2±1.3	119.6±1.2	140.0±1.2	156.8±1.3	<0.001
Urine Cr (mmol/L)	20.0±0.1	13.9±0.1	10.8±0.1	7.7±0.1	<0.001
U [Na^+^]/[Cr] (mmol/mmol)	5.0±0.1	8.9±0.1	13.3±0.1	24.1±0.1	<0.001

Data are expressed as means ± SEM. Abbreviations: BMI, body mass index; obesity, BMI ≥25 kg/m^2^ or more; BP, blood pressure; hypertension, systolic blood pressure ≥140 mmHg or diastolic blood pressure ≥90 mmHg or use of antihypertensive medications irrespective of BP; FPG, fasting plasma glucose; diabetes, fasting plasma glucose ≥7.0 mmol/L or current anti-diabetes medication or a previous diagnosis of diabetes by a physician; LDL-C, low density lipoprotein-cholesterol; TG, triglyceride; eGFR, estimated glomerular filtration rate.

### Prevalence of microalbuminuria according to the estimated 24-h urine Na excretion

The median estimated 24-h urine sodium excretion was 3766 (inter-quartile range 2361–5780) mg. The prevalence of microalbuminuria increased with the 24-h urinary sodium excretion and was highest in the fourth quartile group of the estimated 24-h urinary sodium excretion (11.8%). The trend for the prevalence of microalbuminuria remained after adjusting for age, sex, and related diseases, such as diabetes, hypertension, and obesity (Table 3).

**Table 3:** Prevalence of albuminuria according to the estimated 24-h urinary Na excretion.

	Estimated 24-h urinary Na excretion (mg/day)				*P* for trend	*P* for quartile 3 *vs*. quartile 4
	Quartile 1	Quartile 2	Quartile 3	Quartile 4		
Model 1	5.3	5.7	7.5	11.8	<0.001	<0.001
Model 2	7.0±0.7	6.5±0.7	6.9±0.7	9.9±0.7	0.004	0.003
Model 3	6.8±0.7	6.8±0.7	7.0±0.7	9.8±0.7	0.009	0.005

Model 1, no adjustments;

Model 2, adjusted for age and sex;

Model 3, adjusted for diabetes, hypertension, obesity, and the variables in Model 2.

### Association between the estimated 24-h urinary sodium excretion and albuminuria

In the multiple logistic regression analysis, the second and third quartiles of the estimated 24-h urinary sodium excretion were not associated with the presence of albuminuria with the first quartile as a control. However, the fourth quartile was significantly associated with the presence of albuminuria (odds ratio (OR) 1.61, 95% CI 1.17–2.21, *p* = 0.003) after adjusting for age, sex, diabetes, obesity, and hypertension. This remained clear even after adjusting for several factors and excluding patients with an eGFR <30 ml/min. (Table 4).

**Table 4:** Odds ratio for albuminuria in each quartile of estimated 24-h urine sodium with the first quartile as a control.

	Model 1		Model 2		Model 3	
	OR (95% CI)	*P*	OR (95% CI)	*P*	OR (95% CI)	*P*
		0.001		0.005		0.005
1^st^ quartile	-	-	-	-	-	-
2^nd^ quartile	0.98 (0.70–1.38)	0.917	1.05 (0.74–1.50)	0.771	1.00 (0.68–1.46)	0.992
3^rd^ quartile	1.12 (0.81–1.55)	0.508	1.14 (0.81–1.59)	0.454	1.12 (0.78–1.61)	0.528
4^th^ quartile	1.63 (1.20–1.22)	0.002	1.61 (1.17–2.21)	0.003	1.63 (1.16–2.29)	0.005

Model 1: adjusted for age and sex.

Model 2: adjusted for age, sex, diabetes, hypertension, and obesity.

Model 3: Model 2 after excluding eGFR <60 ml/min/1.73 m^2^ (n = 4964).

After stratification by the presence of obesity (BMI ≥25 kg/m^2^), the fourth quartile of the estimated 24-h urinary sodium excretion in the non-obese group was significantly associated with the presence of albuminuria (OR 1.59, 95% CI 1.08–2.36, *p* = 0.020) after adjusting for age and sex. In the obese group, however, the fourth quartile of the estimated 24-h urinary sodium excretion was not associated with the presence of albuminuria (OR 1.58, 95% CI 0.95–2.61, *p* = 0.079).

## Discussion

In this population-based analysis, a higher salt intake as represented by the estimated 24-h urinary sodium excretion was associated with the presence of albuminuria. This association between salt intake and albuminuria was clear, even after adjusting for age and other factors. These findings suggest that salt intake is an important determinant of albuminuria in the adult Korean population.

Several cross-sectional studies have shown an association between sodium intake and urinary albumin [15]–[19]. The Framingham Offspring Study included 2,700 participants (53% females; median age 58 years) who underwent routine examinations between 1995 and 1998 and showed that the log urinary sodium index was associated positively with the log ACR and the urinary ACR in the fifth quintile of the urinary sodium index was twice as high (95% CI 72–150%) as in the lowest quintile after adjusting for age, sex, diabetes, diuretic use, systolic and diastolic blood pressures, hypertension treatment, serum creatinine, and smoking [15]. Vedovato *et al*. reported that switching from a low to a high sodium diet resulted in increases in blood pressure, body weight, and albuminuria in type 2 diabetics with microalbuminuria (n = 20), but no changes occurred in type 2 diabetics without microalbuminuria (n = 21). The finding of a significant association between sodium intake and albuminuria regardless of blood pressure in several studies suggested that the detrimental effect of sodium is not solely pressure-mediated [17], [20], [21]. Some animal and human studies have shown that salt induced renal injury by increasing the systemic and intraglomerular blood pressures [22], [23] and by a pressure-independent mechanism. A high salt intake was linked to target organ injury, including left ventricular hypertrophy and renal fibrosis [24]. Recently, inflammation has also been suggested to be related to salt intake and albuminuria. In primary hypertensive patients, the urinary sodium intake is correlated with both albuminuria and C-reactive protein, independent of any blood pressure effect [18].

Conversely, a few studies found no association between sodium intake and albuminuria [25], [26]. In 2000, the Nurse’s Health Study (n = 3348) reported that a high dietary intake of animal fat and red meat was associated with the presence of microalbuminuria, while the salt intake was not, although a higher salt intake was related to a decline in the eGFR [25]. When interpreting this study, we should consider that the Nurse’s Health Study population was composed mostly of older Caucasian females (median age 67 years) with relatively high prevalences of hypertension (54.5%) and diabetes (23.7%). The Coronary Artery Risk Development in Young Adults (CARDIA) Study reported that during the 15-year follow-up, poor diet quality and obesity were associated with the development of microalbuminuria after multivariate adjustment, while smoking, low physical activity, fast food consumption, and sodium intake were not associated with the development of microalbuminuria [26]. The reason for the discrepancy regarding the association of sodium intake with albuminuria is not clear. It might have been caused by the difference in population characteristics, including age, obesity, other cardiovascular risks, routine sodium intake in their community, and ethnicity. One of the main limitations of these two studies was the lack of urinary sodium measurements. A well-known issue with dietary assessment instruments is the under-reporting of nutrient intakes [27], [28]. In both studies, the sodium intake was measured using dietary recall. The recall method is less accurate than estimates based on urinary data [29]. The equation that we used is recommended for estimating salt intake by the Japanese Society of Hypertension and has been validated in other studies [30]–[33].

The Prevention of Real and Vascular End-stage Disease (PREVEND) study reported that sodium intake was positively related to the urinary albumin excretion and this relationship was stronger in subjects with a higher BMI in 7,850 Dutch 28–75 years of age [16]. The Reasons for Geographic and Racial Differences in Stroke (REGARDS) study reported that the highest quintile of dietary sodium was associated with an increased risk for albuminuria in obese participants (OR 1.44; 95% CI 1.00–2.07), but not in normal-weight or overweight participants [19]. However, we did not find a more pronounced positive association between sodium intake and the presence of albuminuria in obese participants in our study. The reason for this discrepancy is not clear. The first possibility is the difference in the study populations. In a previous study, we reported that all obesity indices and the presence of obesity were not associated with the presence of albuminuria in this population [11].

There were several limitations to our study. We were not able to evaluate causality due to the cross-sectional design. In addition, the specific drug history, which might affect urinary sodium excretion, was not available. Another limitation was that urinary sodium excretion was not determined by 24-h collection, but by an estimation from a random urine sample. Despite these limitations, with the strength of a nation-wide representative sample and the clear association of the estimated 24-h urinary sodium excretion with the presence of albuminuria, the data suggest that sodium intake is involved in the development of albuminuria.
